# Endometrial microbiome in mares with and without clinical endometritis

**DOI:** 10.3389/fvets.2025.1588432

**Published:** 2025-08-01

**Authors:** Lulu Guo, G. Reed Holyoak, Udaya DeSilva

**Affiliations:** ^1^Department of Veterinary Clinical Sciences, Oklahoma State University, Stillwater, OK, United States; ^2^Department of Animal and Food Sciences, Oklahoma State University, Stillwater, OK, United States

**Keywords:** chronic endometritis, mare, equine, microbiome, uterus, reproductive health

## Abstract

Chronic endometritis (CE) is a major contributor to reproductive failure in mares and in many other mammals. Current diagnostic methods lack sensitivity due to the lack of pathognomonic clinical signs or ultrasound findings. Although microbial involvement was suggested, no definitive causative agents have been isolated, and the few studies conducted are compromised by the dependence on culturable aerobic organisms. This study compares the endometrial microbiomes of 13 healthy and 13 CE-diagnosed mares that were carefully matched to their locations and management. Microbial diversity was significantly reduced in CE mares, indicating dysbiosis. *Burkholderia* and *Chlamydia* were dominant in both groups but significantly more abundant in CE samples. Linear discriminant analysis revealed *Burkholderia*, *Hyphomicrobium*, and *Erwiniaceae* as significantly enriched in CE. Functional pathway analysis showed increased metabolism-related pathways in CE-associated microbiota, while healthy mares exhibited greater microbial richness and functional diversity. These findings underscore microbial imbalance as a potential driver of CE and highlight the utility of sequencing-based microbiome profiling for improved diagnosis and therapeutic targeting in equine reproductive health. This preliminary study contributes to establishing a uterine microbiome reference for mares, with implications for fertility management.

## 1 Introduction

There is strong evidence that chronic endometritis (CE) is an important factor causing female reproductive failure. As a persistent recurrent inflammation of the endometrium, CE disrupts the microenvironment and destroys the commensal coexistence between microorganisms and the host immune system in the uterus (1). Chronic inflammation creates a hostile environment that may be embryotoxic and/or hinders normal placentation of an embryo and its subsequent development. It occurs in various animal species, including humans and mammals such as cows, mares, and bitches. Chronic endometritis refers to an extended duration of inflammation of the endometrium. It can lead to reproductive issues and infertility in affected humans and animals. Diagnosed CE is detected in 30% of infertile women (2), 18.1% of infertile cows (3), 15% in bitches over 8 years of age (4), and from 25 to 60% in infertile mares (5–7). One study of Thoroughbred mares in the United Kingdom reported that 34% of mares diagnosed with infertility had CE (8). Similarly, in Warmblood mares in Germany, 32% of infertile mares had CE, and the severity of the disease correlated with decreased pregnancy rates (9). The American Association of Equine Practitioners (AAEP) lists CE as one of the most common causes of infertility in mares in North America (10), and approximately 28% of clinically normal mares were shown to have subclinical endometritis (11).

Therefore, CE is a leading factor in infertility or reduced fertility in breeding mares and can have a marked economic impact on the equine industry. It can lead to reduced conception rates and an increased number of cycles and treatments required to achieve pregnancy, which result in additional expenses associated with repeated breeding attempts, including veterinary fees, treatment costs, semen collection and processing costs, and transportation expenses. It also results in lower foal production rates, which can directly impact the profitability of breeding operations. Therefore, prevention, early diagnosis, and appropriate treatment are very important.

The diagnosis of CE is hampered by there being no pathognomonic clinical signs or ultrasound findings. In women, it is based on the identification of endometrial stroma’s plasma cells; classic CE diagnostic methods depend on histology (12), but this method is non-specific and dependent on the menstrual cycle’s date when sampling proceeds. In the mare, while endometrial cytology and microbial culture are most commonly used for CE diagnosis (2, 13–15), the most common clinical sign is the presence of luminal fluid within the uterus (16). That is subjective because the normal equine estrous cycle induces endometrial edema and mild luminal fluid accumulation. Microbial culture for the identification of endometrial pathogens concomitant with inflammatory cells on endometrial cytology is the only way to provide objective information for targeted therapy (14). However, endometrial cytology and culture lack sensitivity (17, 18) and endometrial bacterial culture is not always useful since it often has a relatively long turnaround time, and likely not all microorganisms leading to CE are culturable (15). The uterine microbiome refers to the diverse community of microbiota that inhabit the uterus. To increase our understanding of eubiosis compared to dysbiosis within the equine endometrial microbiomic environment, we studied comparable microbiomes of location, diet, and management matched clinically healthy mares and those with diagnosed CE.

While traditional diagnostic approaches for CE, such as bacterial culture and cytology, have been widely used, they suffer from limited sensitivity and often fail to detect the full spectrum of uterine microorganisms. These methods predominantly identify culturable bacteria, overlooking the significant portion of the microbiome composed of unculturable or fastidious species. High-throughput sequencing technologies, such as 16S rRNA gene sequencing, provide a comprehensive and culture-independent approach to characterize the uterine microbiome in greater depth. By capturing the diversity and relative abundance of both cultivable and non-cultivable microbes, these advanced techniques offer new opportunities to unravel microbial dysbiosis associated with CE, improving our understanding of its pathogenesis and potentially guiding more precise diagnostic and therapeutic strategies. Here we provide some preliminary findings of the uterine microbiomes between the healthy mare and symptomatic mare by applying high-throughput sequencing technologies to add insight into diagnosing and possible treatments for CE.

Recent studies have increasingly focused on characterizing the reproductive tract microbiota using culture-independent methods, revealing complex microbial communities that may influence fertility and uterine health (19, 20). Similar investigations in other large animals, such as the donkey (21) and cattle (20), have also underscored the importance of microbial balance in reproductive success. However, despite these advances, comprehensive analyses specifically comparing uterine microbiomes of mares with chronic endometritis to healthy controls remain limited (22, 23). We have previously described the existence of a dynamic microbiome in healthy equine and canine uteri (24, 25). To our knowledge to date, the comprehensive microbiome analysis in CE horses is not well established. These preliminary findings begin the development of an essential data bank.

## 2 Methods

### 2.1 Experimental design and sample collection

A total of 26 mares were selected for this study. Mares ranged from 4 to 18 years of age and did not have recent antibiotic exposure. All the enrolled mares are from Oklahoma, but from three different ranches. Five of the CE and five healthy mares were from a facility in South-central OK. Four samples in each group were from a facility in central OK, and the rest of the samples were from mares from the Oklahoma State University Veterinary Medicine Ranch. These mares were long-term occupants of their respective resident facilities with similar diets. Each location contributed equal numbers of normal and CE mares to counter the possible influence of the sample source. In total, 13 healthy mares with normal reproductive histories and no clinical signs were recruited as the healthy group, which was matched by location and diet to 13 mares previously diagnosed with CE and assigned to the CE group in this study. To make the diagnosis of CE, each mare had greater than two unsuccessful breeding attempts with failure to have a diagnosed pregnancy and associated examination findings of uterine inflammation. Each mare underwent serial reproductive tract examinations consisting of transrectal palpation and ultrasonographic examination. When the presence of uterine luminal fluid and associated inflammatory endometrial edema was detected, the examination was followed by endometrial cytology and bacteriological culture to confirm the diagnosis of endometritis. These samples were obtained when the mare presented for examination in late diestrus and early estrus as part of routine breeding management on each farm. All mares were beyond their postpartum period.

Endometrial microbial lavage samples were collected with a sterile triple-guarded system to minimize the risk of vaginal microbial contamination as described in equine microbiome studies (24). The perineum and external genitalia were cleaned three times with an iodophor scrub and alcohol to minimize external contamination. Sterile obstetrical lubricant was used on a sterile disposable speculum before entering the reproductive tract. Sterile obstetrical sleeves were applied to protect and guide the speculum and catheter through the cervix. A measure of 150 mL of saline was infused, agitated within the uterine lumen, and after 30s of contact with the endometrium, 45 mL of lavage was decanted and collected midstream. All samples were stored on ice temporarily and transported to the laboratory within 2 h. The samples were processed in the laboratory immediately, within 2 h after sampling.

### 2.2 Negative control

Negative controls were processed in the same way as regular samples. Each negative control was collected on site at the same time as sample collection and processed alongside the samples, with the only difference being that they were not flushed through the reproductive tract. DNA isolation and 16S rRNA amplification were conducted with the two sample groups together to avoid the potential unnecessary impact on external conditions caused by human factors. All the laboratory reagents were the same. Sample treatment and subsequent sequence analysis were performed by a single investigator. Totally, three negative control samples were collected and used in this study. All negative control samples failed in the amplification and sequencing steps.

#### 2.2.1 DNA extraction

Lavage samples (45 mL) were spun down at 7000 rpm for 15 min at 4°C. DNA of all CE, healthy, and negative control samples was extracted in a controlled environment using a QIAamp DNA MINI KIT (Qiagen, Germantown, MD) following the manufacturer’s protocols. Quality and quantity of DNA were measured with a spectrophotometer (NanoDrop 1,000, Thermo Fisher Scientific, Wilmington, MA).

### 2.3 16S rRNA gene amplicon sequencing

The PCR amplification of extracted DNA and 16S amplicon sequencing was conducted by a universal primer set of 515F (5-GTGCCAGCMGCCGCGGTAA-3) and 806R (5-GGACTACHVGGGTWTCTAAT-3), which targets the bacterial 16S V4 hypervariable region (~250 bp) by using Phusion^®^ High-Fidelity PCR Master Mix (New England Biolabs). Barcode sequences were carried out in the forward primer.

The PCR products were verified by 1.5% agarose gel electrophoresis and extracted using a Qiagen^®^ Gel Extraction Kit (Qiagen, Germantown, MD). The NEBNext^®^ UltraTM^®^ DNA Library Prep Kit (New England Biolabs, Ipswich, MA) was used in library preparation for Illumina. Qubit 3.0 fluorometer and Q-PCR were used for qualification and quantitation, respectively. Subsequently, an Illumina HiSeq 2,500 platform was used for sequencing the library. Purified amplicons were pooled and generated into paired-end sequence reads on an Illumina HiSeq 2,500 (Illumina, San Diego, CA).

### 2.4 Bioinformatic and statistical analysis

The sequences were mainly analyzed using the latest QIIME2 (26). Data preprocessing is based on the overlap between reads. The paired-end sequence data obtained by HiSeq sequencing were merged into sequence tags. Then, quality control filtering was performed to detect the reads’ quality and the merge effect. There are mainly three steps. First, the reads of each sample were merged by overlapping, and the merged sequence obtained was the original tags data (Raw Tags). Second, we filtered the merged raw tags to obtain high-quality tag data. Finally, chimeras were removed to obtain the final valid data.

UCLUST in QIIME2 was used to cluster the tags at a similarity level of 97% to obtain OTUs, and the OTUs were taxonomically annotated based on the Silva (bacteria) and UNITE (fungi) taxonomic databases. In this step of QIIME2, there are mainly two methods, DADA2 (27) and Deblur (28). DADA2 was used for clustering, as it was proven to be superior to other clustering methods (29). Compared with QIIME’s UPARSE clustering method, the current DADA2 method will only remove noise and chimeras, but cluster by similarity. It has more accurate analytical results than the previous generation. The main function of DADA2 is to remove low-quality sequences and chimeras and regenerate the OTU table, which is now called the feature table. Because the clustering method is no longer used, the feature table is equivalent to the OTU table with 100% similarity in the QIIME era.

All statistical analyses were performed in R language. Alpha diversity was analyzed using Shannon indexes, and a Student’s t-test was performed to illustrate the differences in the indexes between the two groups. Differences in community composition were demonstrated by the Wilcoxon test. The beta diversity was analyzed by non-metric multi-dimensional scaling (NMDS) using the ape software package in R language, and the differences between groups and within groups were evaluated by the analysis of similarity (ANOSIM) statistical test. The linear discriminant analysis (LDA) effect size was applied to identify differentiated bacteria between the two groups. Tax4Fun software was utilized to infer the functional gene composition in the sample by comparing the species composition information obtained from the 16S sequencing data. Differential abundance testing was performed using linear discriminant analysis effect size (LEfSe), with an LDA score threshold of >2.0 to identify taxa significantly associated with either group. Multiple testing corrections were applied through false discovery rate (FDR) adjustment to control for type I error. Additionally, the Metastats method was used to test for significant differences in species abundances, also incorporating FDR correction.

**Figure 1:**
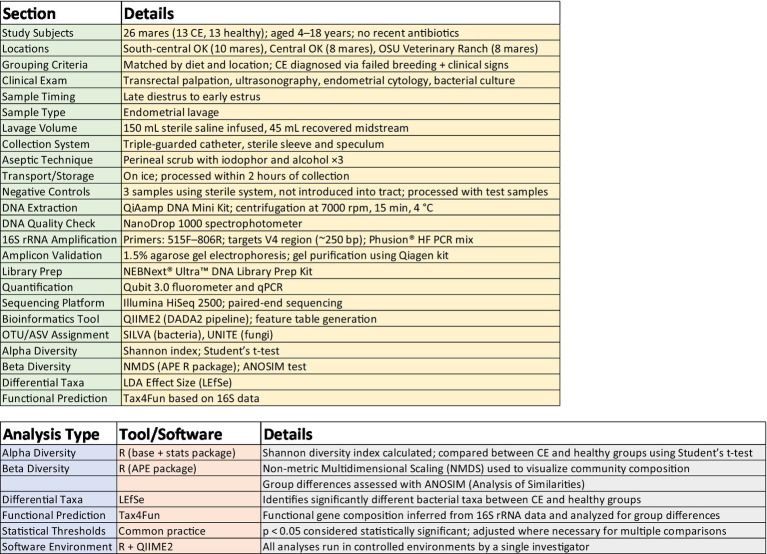
Experimental workflow and software utilized.

The experimental workflow and a summary of bioinformatic software utilized for various analyses are depicted in Figure 1.

## 3 Results

Negative control samples for both CE and healthy samples failed in the two-step PCR amplification protocol for the 16S rRNA V4 region, which indicates that bacteria in the negative control samples were negligible (30). As mentioned above, each location contributed equal numbers of normal and CE mares to counter the possible influence of the sample source. Rarefaction curves performed on CE and healthy groups indicated that the sequencing depth was sufficient to cover the overall bacterial diversity in all samples (data not shown). A total of 2,060,513 sequence reads were obtained from 13 healthy mare samples, and 1,913,005 sequence reads were retained after removing the host and low-quality reads. More than 90% of the reads passed the denoise and chimera removal. Totally, 426,946 OTUs were obtained. 267,802 OTUs in the CE mare group and 192,765 OTUs in the healthy mare group were used in downstream analysis.

**Figure 2:**
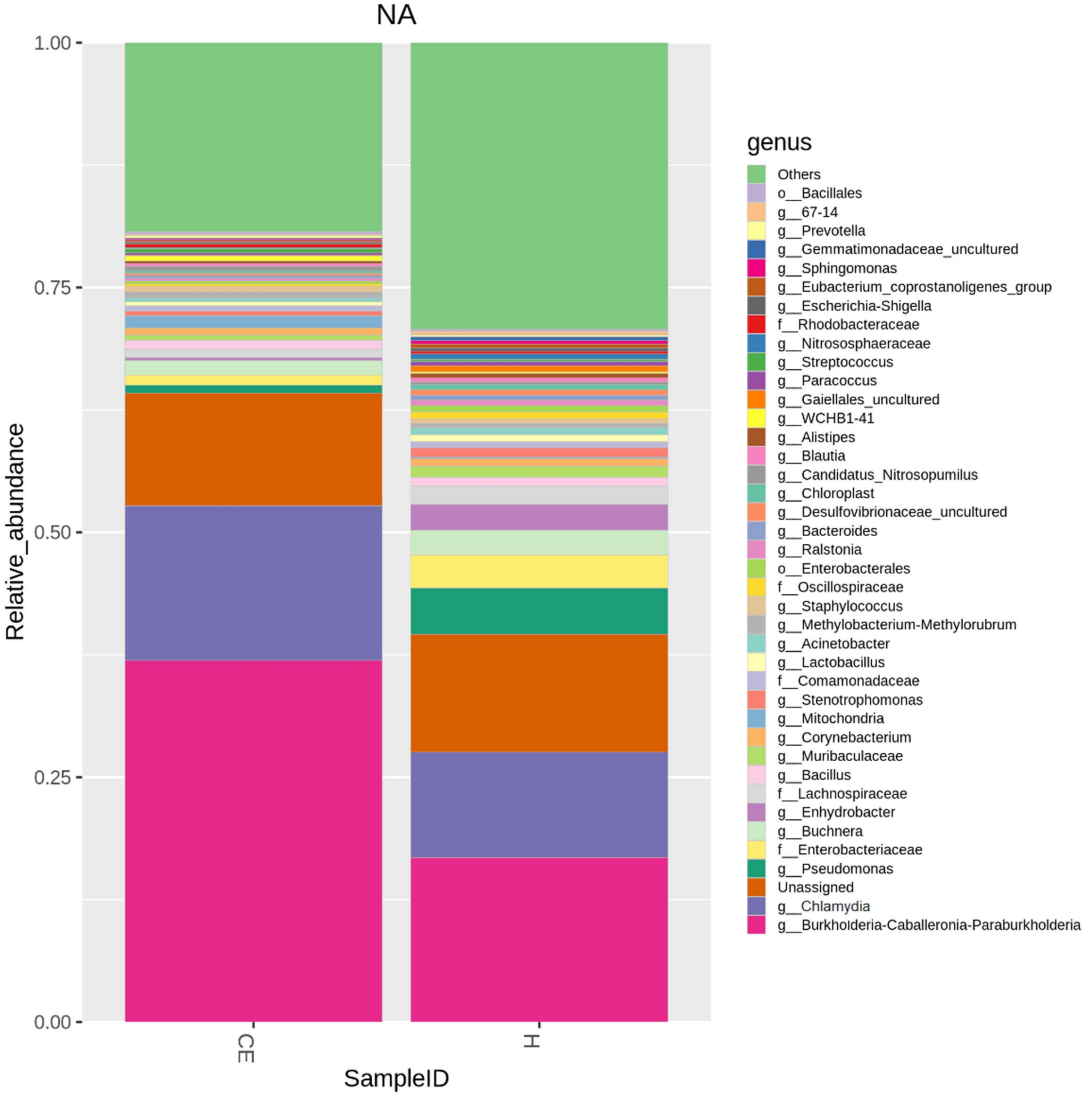
Microbiome differences between healthy and CE groups: relative abundance of key genera: among the microbiome differences observed between the healthy and CE groups, Burkholderia and Chlamydia were the most abundant genera in the endometrial microbiomes of both groups. However, the Wilcoxon test revealed that Burkholderia (*p*-value = 0.024665 < 0.05) and Chlamydia (*p*-value = 0.05 ≤ 0.05) exhibited significantly higher relative abundances in the CE group compared to the healthy group. Other genera with lower relative abundance included Pseudomonas, Enterobacteriaceae, and Buchnera.

**Figure 3:**
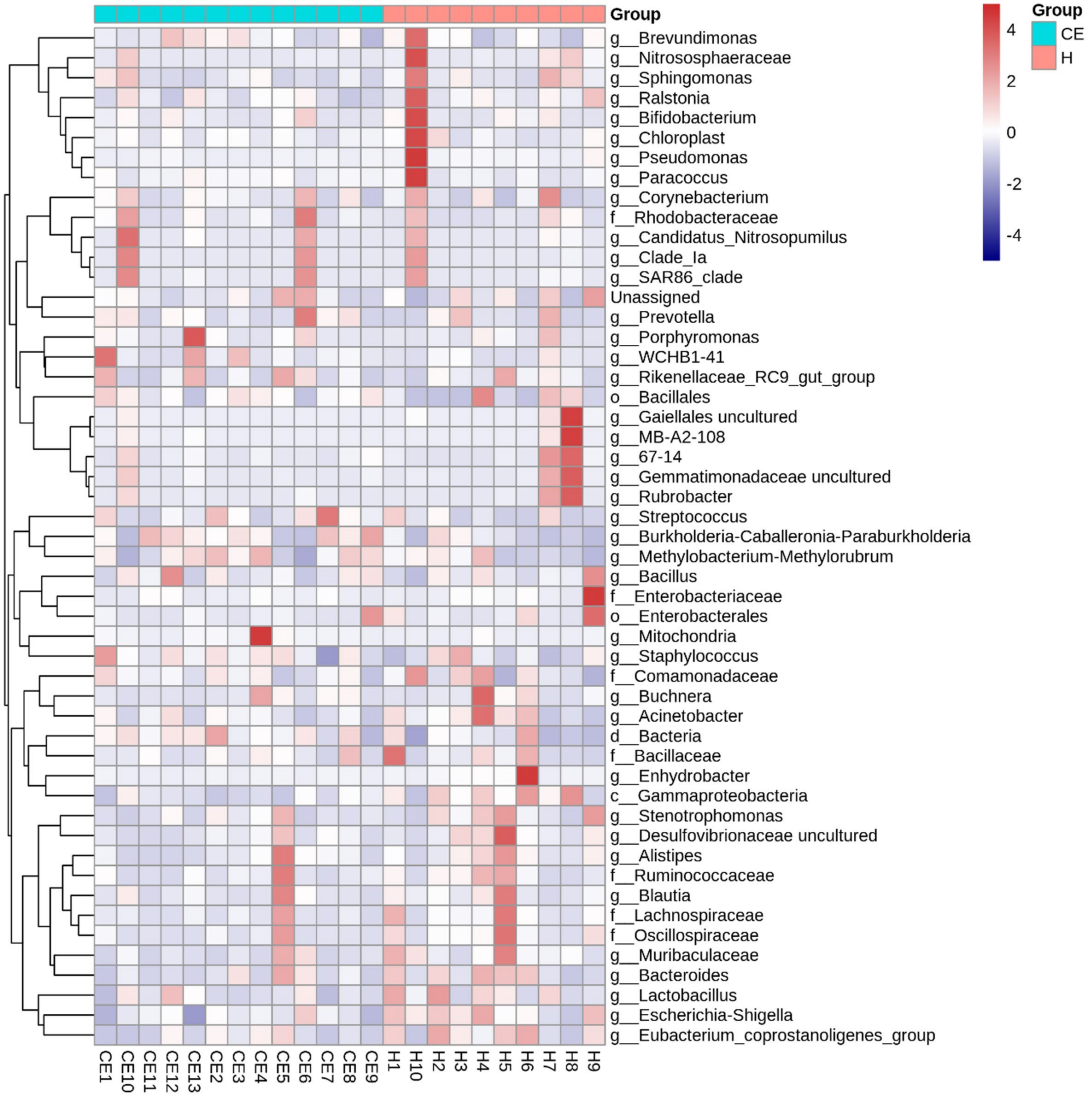
Heatmap of the top 50 bacterial genera in uterine samples from healthy mares and mares with chronic endometritis (CE). The heatmap of the top 50 genera reveals reduced clustering in the CE group compared to the healthy group, indicating distinct microbiome profiles between the two groups. Genera were selected based on the highest mean relative abundance across all samples. Abundance values were normalized by Z-score transformation per genus to facilitate visual comparison across samples. Color intensity reflects standardized abundance, with red indicating higher and blue indicating lower relative abundance.

**Figure 4:**
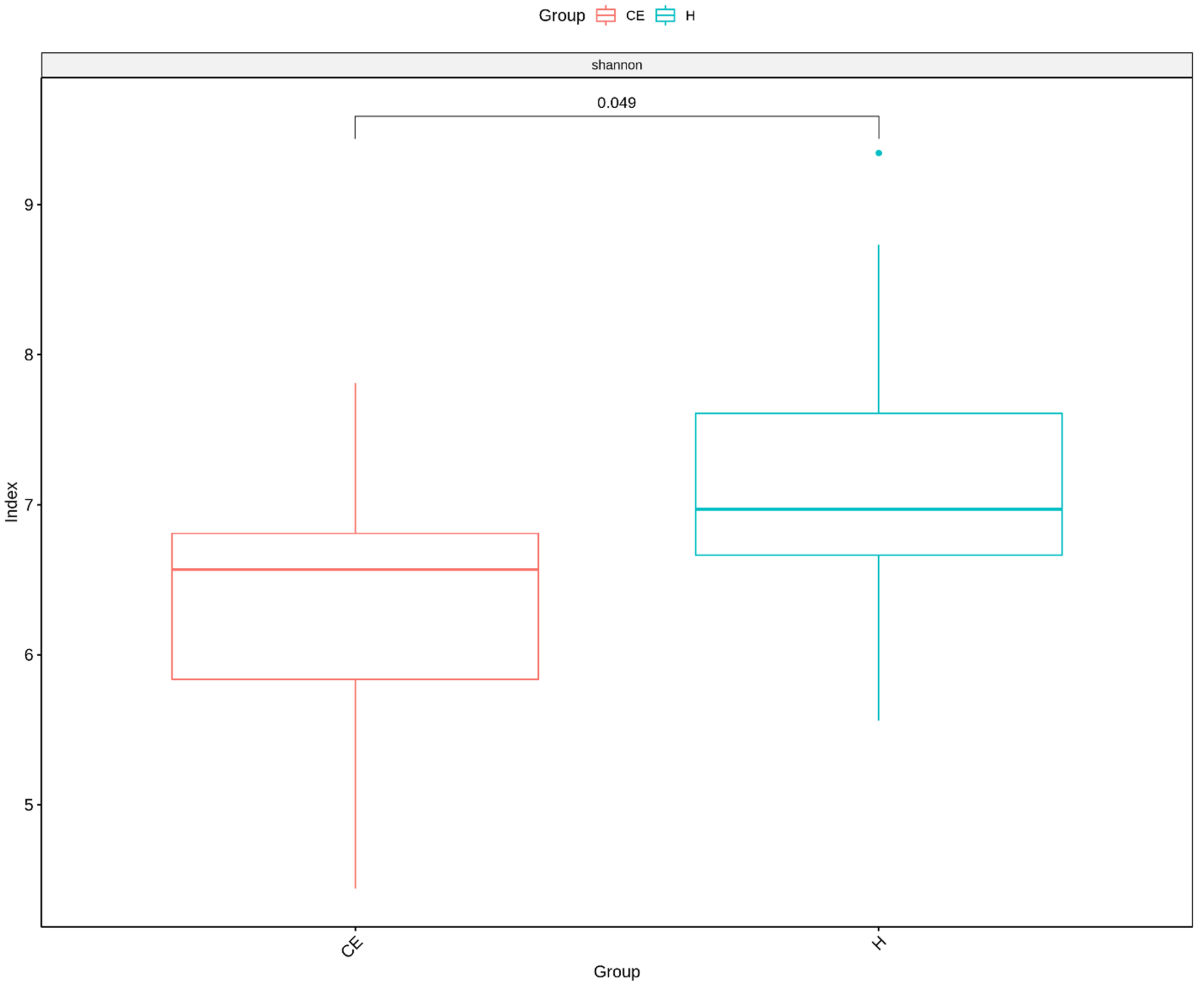
The Shannon index revealed significant differences between the two groups (*t*-test, *p*-value = 0.049 < 0.05). The healthy group exhibited significantly higher richness and evenness compared to the CE group.

**Figure 5:**
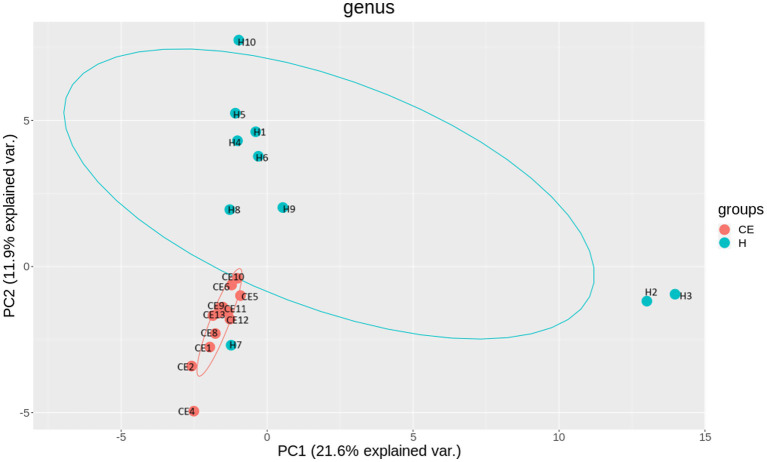
Beta diversity reflects the similarity or dissimilarity of microbial communities between groups. Principal Coordinate Analysis (PCoA) based on Bray-Curtis dissimilarity revealed two distinct clusters, indicating differences in community composition between the healthy and CE groups. Specifically, In the CE group, mares 1 and 8 are from the OSU ranch; mares 2, 4, 6, and 10 are from central Oklahoma; mares 5, 9, 11, 12, and 13 are from south-central Oklahoma. In the healthy (H) group, H7 is from the OSU ranch; H5, H8, H4 and H10 are from central Oklahoma; H1, H2, H3, H6, and H9 are from south-central Oklahoma.

**Figure 6:**
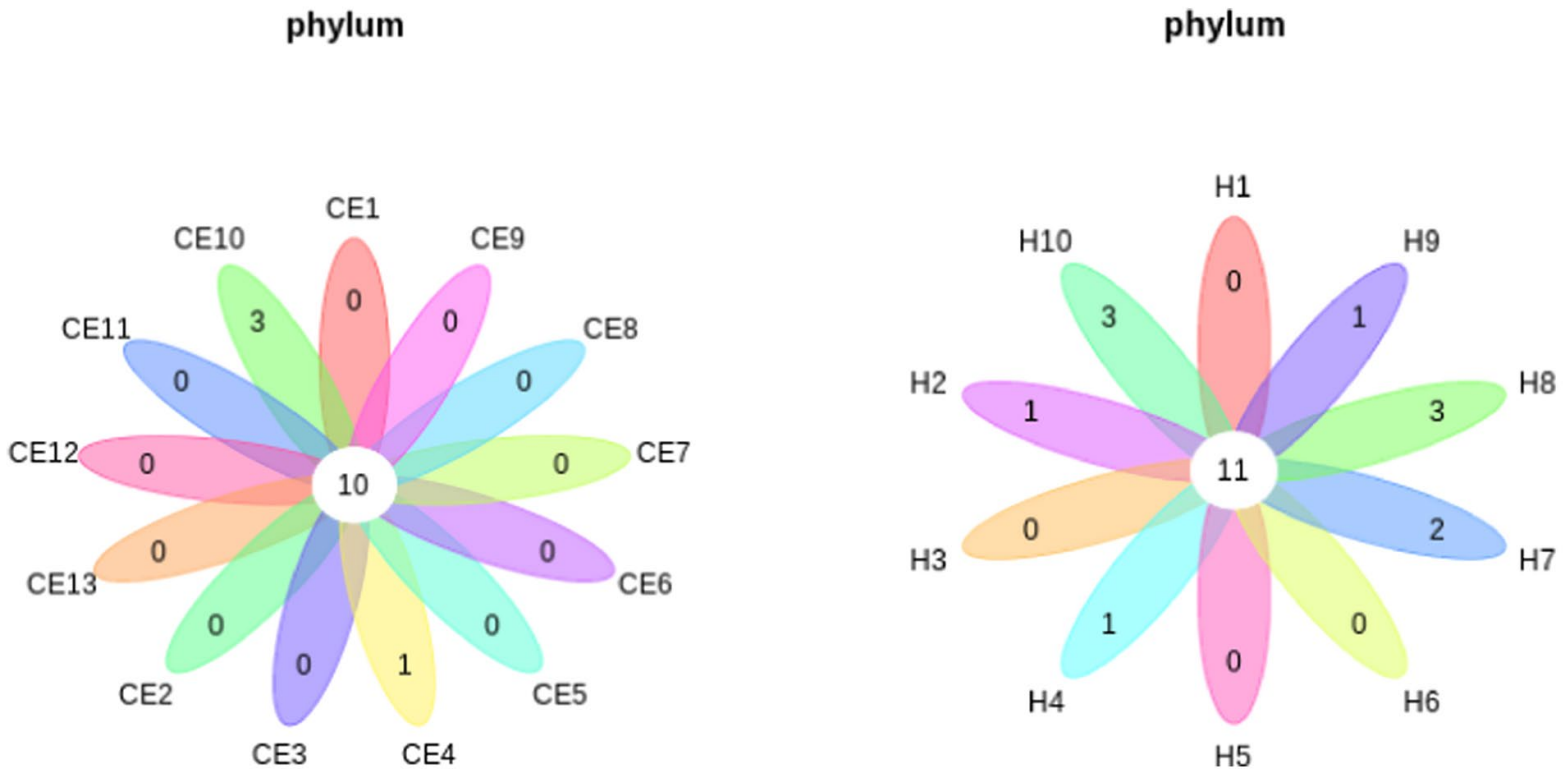
Petal diagrams show the common and unique microbial diversity across different groups. The central circle represents the number of phyla shared by all samples, while the numbers on the petals indicate the number of phyla unique to each group. Unique phyla found only in the CE group include Halobacterota, uncultured delta Proteobacterium Sva0485, and WPS-2, a phylum closely related to Chloroflexi, Armatimonadetes, and Dormibacteria. Unique phyla in the healthy group include Acetothermia, Caldatribacteriota, Deferribacterota, Margulisbacteria, and Fibrobacterota.

Among the microbiome differences between the healthy group and the CE group, *Burkholderia* and *Chlamydia* were the most abundant genera in the endometrial microbiomes of both groups (Figure 2). However, the Wilcoxon test showed *Burkholderia* (*p*-value = 0.024665 < 0.05) and *Chlamydia* (*p*-value = 0.05 ≤ 0.05) in the CE group had significantly higher relative abundance than in the healthy group. The rest of the genera with lower relative abundance were *Pseudomonas*, *Enterobacteriaceae,* and *Buchnera*. There was a significant difference (*p*-value = 0.00262 < 0.05) between the two groups based on the top 50 genera by the Wilcoxon test. A total of 608 genera were shared by the two groups. A total of 292 and 211 exclusive genera were identified in the healthy group and CE group, respectively. The heatmap of the top 50 genera showed that the CE group had decreased clustering compared to the healthy group (Figure 3). The alpha diversity (Figure 4) in the Shannon index was used to calculate the richness and evenness in the microbiome and indicated significant differences between the two groups via t-test (*p*-value = 0.049 < 0.05). The healthy group was statistically higher in richness and evenness compared to the CE group. Unlike the alpha diversity analysis, beta diversity is used to reflect the similarity or dissimilarity of the microbial communities between groups. Principal coordinate analysis (PCoA) with Bray–Curtis dissimilarity showed two distinct clusters in community composition (Figure 5). A petal diagram was drawn to analyze the common and unique information about microbial diversity between the groups (Figure 6). The central circle of the petal diagram represents the number of phyla shared by all samples, and the numbers on the petals represent the number of phyla unique to the group of samples. The unique phyla found only in the CE group are Halobacterota, the uncultured delta proteobacterium Sva0485, WPS-2 (a phylum that exhibits high genetic similarity to Chloroflexi), Armatimonadetes, and Dormibacteria. The unique phyla in the healthy group were Acetothermia, Caldatribacteriota, Deferribacterota, Margulisbacteria, and Fibrobacterota. As expected, the CE group mares had very few phyla that were unique to individual animals.

**Figure 7:**
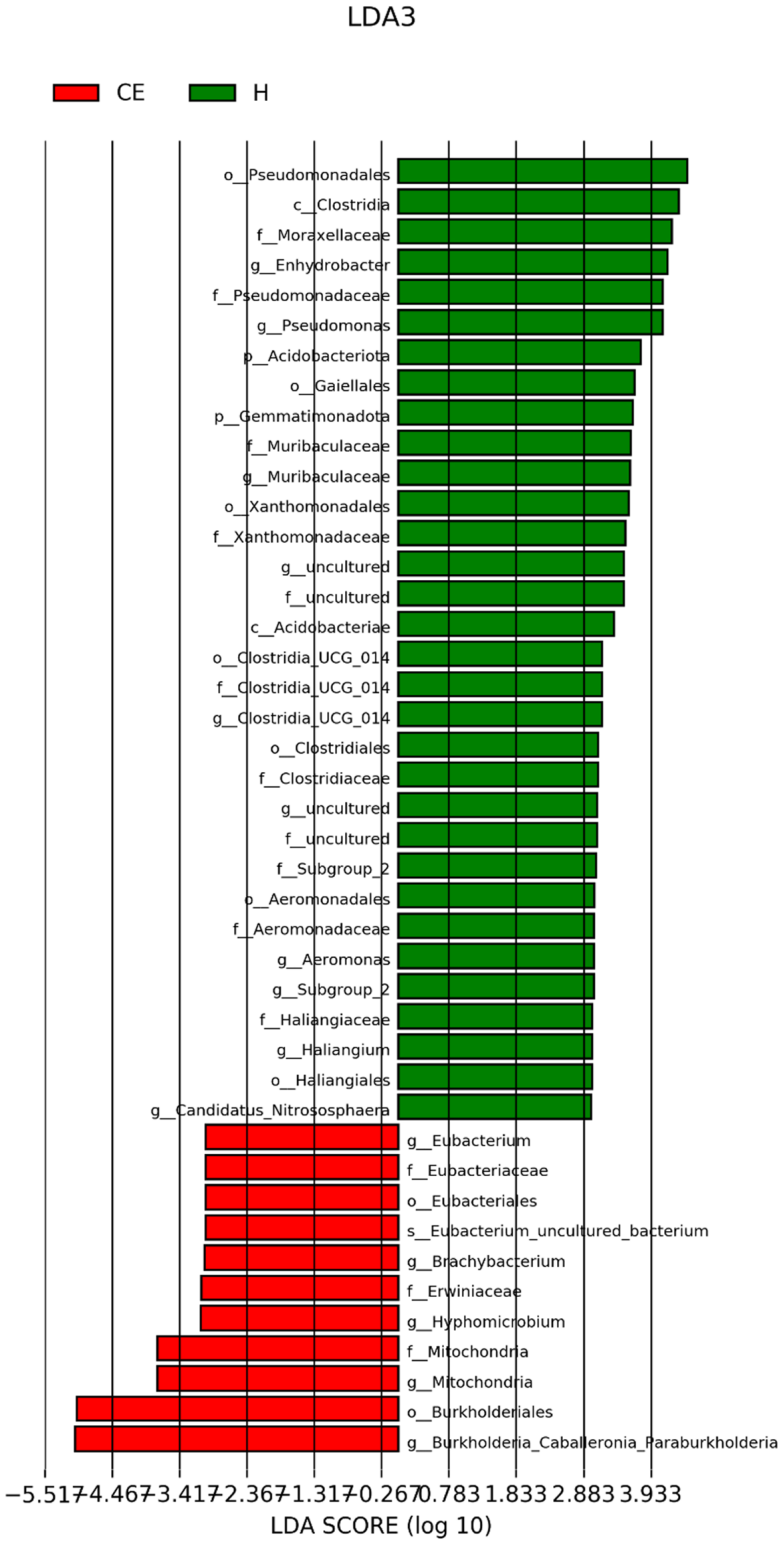
LEfSe (Linear Discriminant Analysis Effect Size) with a linear discriminant analysis (LDA) score > 2 was applied for filtering species with significant differences between groups. Burkholderia (genus), Burkholderiales (order), Hyphomicrobium (genus), and Erwiniaceae (family) showed significant associations with CE mares, exhibiting higher LDA scores.

**Figure 8:**
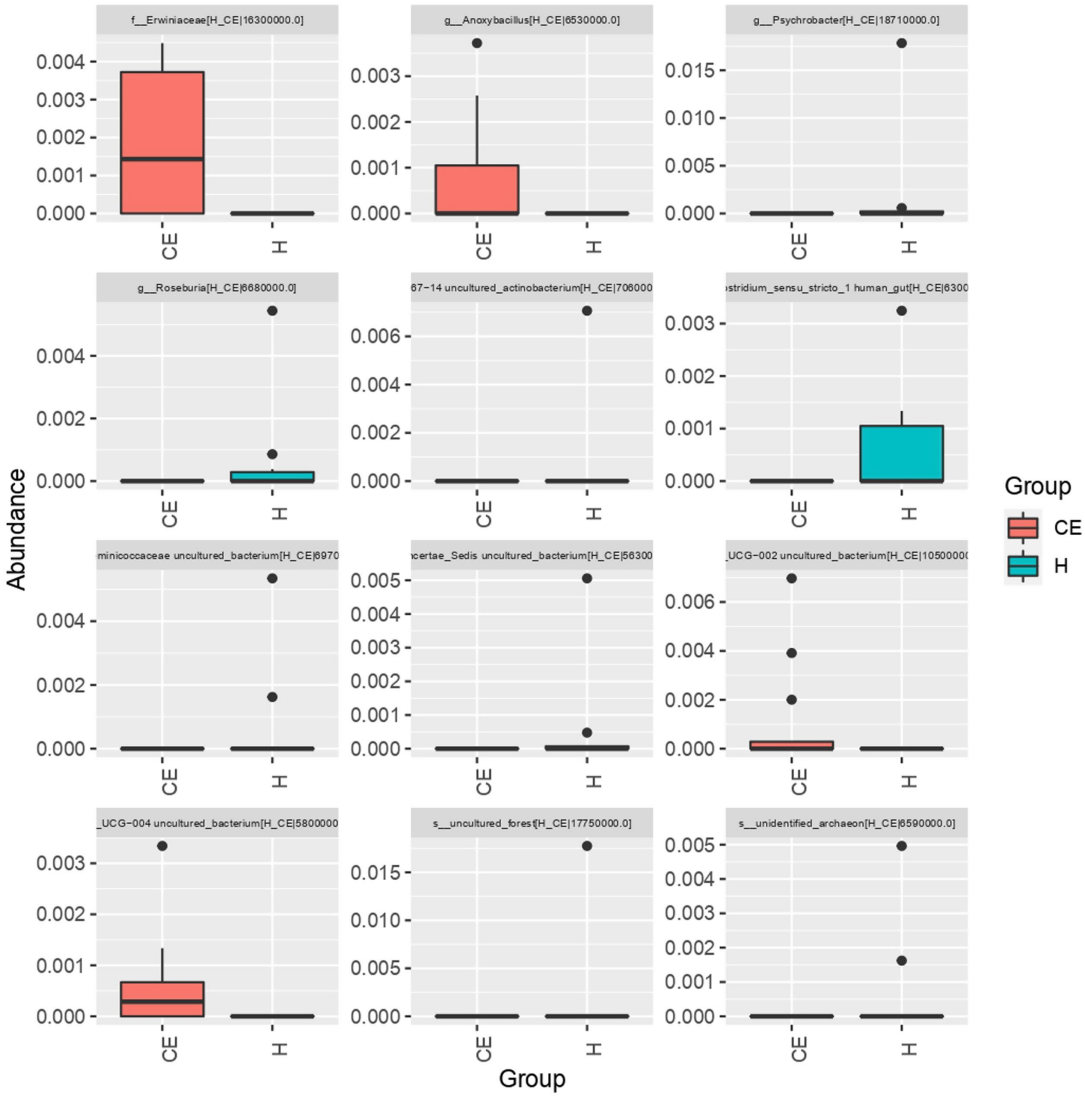
The boxplot of abundance distribution for different species between groups revealed that Erwiniaceae (family), Anoxybacillus (genus), and two other uncultured species were significantly more abundant in the CE group compared to the healthy group. In contrast, Clostridium species and Roseburia genus were more abundant in the healthy mares.

**Figure 9:**
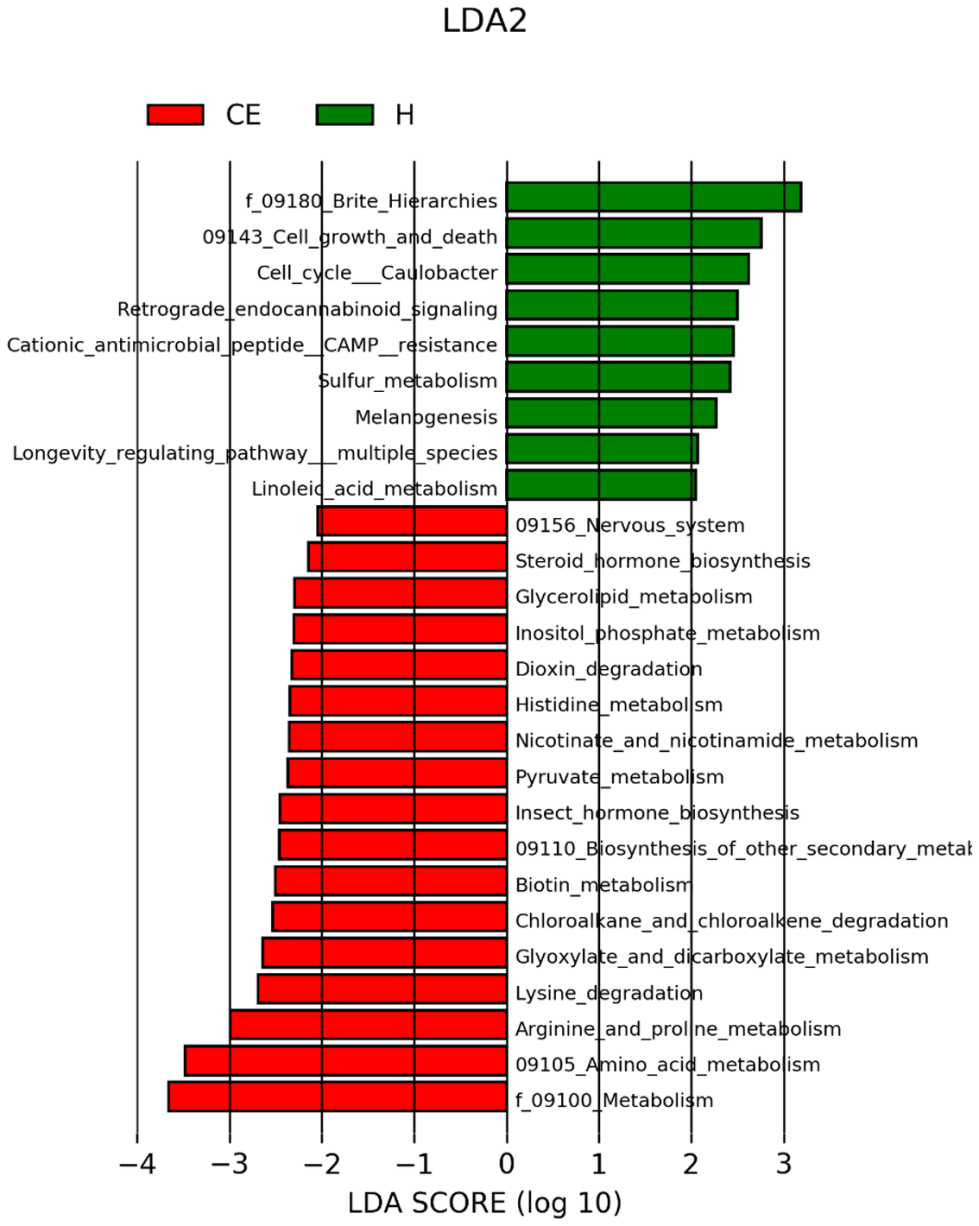
Comparison of predicted KEGG metabolic pathways in the uterine microbiomes of healthy mares and mares with chronic endometritis (CE), based on Tax4Fun functional inference. Welch’s t-test with 95% confidence intervals was performed in STAMP to compare functional pathway abundance between groups. Blue bars represent the healthy group, and yellow bars represent the CE group. Microbial communities in CE mares exhibited lower richness in Brite Hierarchies pathways but showed a higher relative abundance of metabolic pathways compared to the healthy group.

To filter species with significant differences between groups, LEfSe with a linear discriminant analysis score of > 3 was applied to confirm the differentially abundant taxa (Figure 7). Burkholderia (genus), Burkholderiales (order), Hyphomicrobium (genus), and Erwiniaceae (family) had a significant association with CE mares with higher LDA scores. To study the species with significant differences between groups, the Metastats method was used to perform hypothesis testing on seven different abundance level data between groups to obtain the *p*-value, and to screen the species with significant differences according to the p-value. Boxplot of the abundance distribution of different species between groups indicated Erwiniaceae at the family level, Anoxybacillus at the genus level, and two other uncultured species were significantly higher in the CE group than the healthy group. Clostridium species and the Roseburia genus were richer in healthy mares (Figure 8). The functional profile of the endometrial microbiome was compared between the CE and healthy groups by KEGG pathway analyses (Figure 9). Welch’s t-test with 95% confidence intervals was used through STAMP. Microbiota in the CE group showed significantly less richness than the healthy group in pathways of the Brite Hierarchies factors. In addition, metabolism was significantly more abundant in the CE group compared to the healthy group.

## 4 Discussion

There is growing interest in the role of the mare’s uterine microbiome in the development and progression of chronic endometritis. Because there is a lack of sensitivity with current diagnostic techniques, the combination of culture and cytology at only 42% (17), research in this area is needed to lead to improved diagnosis and treatment strategies for CE in mares. This study investigated the differences in the uterine microbiome between relatively small groups of mares with CE and clinically healthy mares in order to better understand the potential microbial interactions and causes of the disease.

Although the most abundant microbial compositions of the two groups were similar, the ratio of bacteria was different. The composition in the healthy group was obviously richer than the CE group, which indicates that the diversity of the microbiome helps maintain local health. The lower richness and evenness in CE is a classic sign of dysbiosis (31) and has been observed in cattle as well (32). In healthy mares, the uterine microbiome is typically high in diversity, which helps to maintain a healthy environment in the uterus. The imbalance of certain bacteria may consume local resources and otherwise suppress resident microbes, thereby decreasing the overall diversity of the microbiomes and inducing dysbiosis. These observations suggest that dysbiosis is the pathogenesis of CE.

Burkholderia-caballeronia-paraburkholderia was the most abundant genus in the CE group, and Burkholderia species can include pathogenic strains. However, it is important to note that Burkholderia, Caballeronia, and Paraburkholderia are three genera of bacteria within the Burkholderiaceae family. These genera are closely related and share similarities in their genetic and phenotypic characteristics, and are often clustered together. They should be further reclassified based on genomic and phylogenetic analysis to better reflect their evolutionary relationships. Since there is insufficient data that could be used in the uterine region, most of the sequences cannot be classified as accurate genera. We expect this study to contribute to reclassifying these bacteria into different genera, reflecting advances in molecular techniques and a better understanding their evolutionary relationships. Reclassification will help in delineating the broadly diverse Burkholderiaceae family and providing a more accurate taxonomy for these bacteria. Interestingly, Burkholderia species have been isolated from the uteri of both sows and women with reproductive failure (33, 34). The significant increase in the Burkholderia cluster in CE suggests it may play an important role in the pathogenesis of CE.

Chlamydia is a type of bacteria that can cause infections in various species. Chlamydia species are known to cause reproductive tract infections in some animals, such as cats, dogs, and ruminants (35–37), but their involvement in chronic endometritis is not well-documented. Chlamydia infections in horses can occur, but they are usually associated with respiratory or ocular diseases (38, 39). Chlamydia is often associated with a sexually transmitted pathogen with ascending infection from the cervix to the endometrium (40). The significant presence of Chlamydia in both healthy and CE animals in this study is surprising, as our previous studies failed to identify significant presence in the uteri of mares selected from various geographical regions (24). This study could draw attention to Chlamydia’s role in the reproductive tract, either as a commensal or pathobiont.

We had identified *Pseudomonas* contributing as much as 27% of uterine microorganisms in healthy mares from the lower midwestern states of the United States in our previous studies (24). Their presence was masked by the dominance of Burkholderiaceae and Chlamydia among healthy animals in this study, and was further reduced in the CE group.

Interestingly, Streptococcus species and *Escherichia coli* are often assumed to have higher levels of pathogenic bacteria in mares with CE. It is probably because routine cultural methods are used to find the targeted bacteria in CE. However, with next-generation sequencing, we obtain relevant information on the many unculturable bacteria and clearer insights into the various roles in dysbiosis versus normobiosis.

Overall, these findings suggest that dysbiosis, or an imbalance in the uterine microbiome, is a contributing factor to the development of CE in mares. With increased understanding of the complex interactions between the mare’s uterine microbiome and the development of CE, the development of new diagnostics specific to the unique changes within the microbiome is possible. Furthermore, being able to discern the induction of dysbiosis can lead to therapeutic preventatives to the disease without the use of systemic or intrauterine antibiotics. This study made a preliminary analysis of the differences between CE mares and healthy individuals. It will enable researchers to study and characterize these bacteria more effectively, including their pathogenicity, ecological roles, and potential mechanisms in CE. Prevention of CE is critical, but how to avoid the risk factors that may lead to dysbiosis and to achieve optimal reproductive health outcomes still requires extensive research. This paper summarized the relationships in the microbiome between a group of CE mares and healthy mares and aimed to provide initial information on the differences.

## Data Availability

Raw sequencing data used in this study is available at Sequence Read Archive (SRA) at ncbi.nlm.nih.gov under the accession number PRJNA1214833.
